# “Like a wake-up call for humankind”: Views, challenges, and coping strategies related to public health measures during the first COVID-19 lockdown in Thailand

**DOI:** 10.1371/journal.pgph.0000723

**Published:** 2022-07-08

**Authors:** Bhensri Naemiratch, Mira Leonie Schneiders, Tassawan Poomchaichote, Supanat Ruangkajorn, Anne Osterrieder, Wirichada Pan-ngum, Phaik Yeong Cheah

**Affiliations:** 1 Mahidol Oxford Tropical Medicine Research Unit, Faculty of Tropical Medicine, Mahidol University, Bangkok, Thailand; 2 Nuffield Department of Medicine, Centre for Tropical Medicine & Global Health, University of Oxford, Oxford, United Kingdom; 3 Nuffield Department of Population Health, Ethox Centre, Big Data Institute, University of Oxford, Oxford, United Kingdom; 4 The SoNAR-Global Network, Mahidol University, Bangkok, Thailand; 5 Department of Tropical Hygiene, Faculty of Tropical Medicine, Mahidol University, Bangkok, Thailand; Emory University, UNITED STATES

## Abstract

Following the first Thai COVID-19 case in January 2020, the Thai government introduced several non-pharmaceutical interventions (NPIs) in March 2020 (e.g., contact tracing, travel restrictions, closure of businesses, curfews, stay at home orders) to control COVID-19 transmissions. This study aimed to understand the views and experiences of a small number of Thai residents related to public health measures implemented during the first COVID-19 wave in Thailand. A total of 28 remote in-depth interviews with Thai residents (18–74 years old) were conducted between 8 May and 21 July 2020. Interviews were audio recorded, transcribed, and analysed using thematic analysis based on the Framework Method. Our results describe participants’ views, challenges, and coping strategies relating to COVID-19 restrictions. Most participants expressed support for the introduction of strict public health measures, while some criticized lacking enforcement or rational of certain measures. Participants identified four major challenges, namely financial hardship; social isolation and loneliness; stigma and shaming; and fear of COVID-19 infection. Strategies adopted to address these challenges included practical coping strategies (e.g., reducing risks and fear of COVID-19 infection; mitigating financial, social, and mental health impacts), and embedded socio-cultural ways of coping (e.g., turning to religion; practicing acceptance; kindness, generosity and sharing (‘*Namjai’*); ‘making merit’ (‘*Tham-bun’*)). The challenges identified from this study, in particular the role of stigma and discrimination, may be relevant to other infectious disease outbreaks beyond COVID-19. Findings from this study underscore the need for policies and interventions that mitigate the negative impacts of NPIs on the public, particularly on vulnerable groups, and highlight the importance of considering socio-cultural context to support community resilience in times of crisis. Our findings remain relevant in light of low COVID-19 vaccine availability and the potential need to implement further public health restrictions in Thailand and elsewhere against COVID-19 or future infectious disease threats.

## Introduction

Thailand is a predominantly Buddhist, upper-middle income country in Southeast Asia [1]. With a population of 67 million, the Thai economy is primarily based on agriculture (8% GDP), industry (34% GDP) and services (58% GDP), where tourism represents one of the most important service sectors driving the Thai economy [2, 3]. Thailand’s first COVID-19 case was reported on 13 January 2020 and by late January, Thailand reported 19 confirmed cases [4]. On 12–13 March 2020, two COVID-19 outbreak clusters were detected in a Thai boxing stadium and several nightclubs, resulting in the rapid spread of cases [5]. Subsequently, a National Emergency Decree was issued on 26 March 2020, authorizing government agencies to enforce specific actions necessary to reduce viral transmission and control the epidemic. The initial restrictions included closure of non-essential businesses, and schools, and prohibiting travelers from entering Thailand except for Thai citizens, people working in shipping businesses, diplomats, or representatives of international bodies working in Thailand [6–8]. The public was requested to remain inside their homes and to limit social contacts. Additionally, on 3 April 2020, a nationwide curfew was announced, with a stay-at-home order implemented between 10 pm and 4 am. A self-report, online contact tracing application “Thai Chana” was adopted to facilitate contact tracing [9], with quick response (QR) code scanning mandated for entering shops, malls, restaurants and public transport. After 9 April, COVID-19 case numbers fell to below 100 per day. With no new cases reported between mid-July 2020 to mid-December 2020, restrictions were gradually lifted again. However, Thailand experienced a second, more severe COVID-19 wave between 18 December 2020 and 27 February 2021 [7], with a seven-fold increase in cases compared to the first wave, triggered by outbreak clusters among migrants working in crowded conditions, including in factories and seafood markets [7].

In the period between April and October 2021, Thailand experienced its third wave, following the rapid spread of the Alpha and Delta variants, resulting in high cases and deaths (with 20,000 new daily cases at its peak in early August and an estimated total of 312 daily deaths by mid-August) [8]. During the third wave, the strict public health measures implemented during the first wave when this study was conducted were largely reintroduced, as the COVID-19 vaccination coverage nationwide had only reached about 50% [10], highlighting the continued importance of relying on public health measures to control COVID-19 infections.

Non-pharmaceutical interventions (NPIs) have been essential to mitigate the impact of COVID-19 in the absence of widely available vaccines and pharmaceutical treatments [11, 12]. NPIs, also referred to in this paper as public health measures, include isolation of sick individuals, quarantine of exposed individuals, social (‘physical’) distancing, contact tracing, movement and travel restrictions, and personal protective measures (e.g., hand hygiene, wearing face masks). Research, including studies conducted in Thailand, indicates that NPIs are effective to contain viral transmission and ease pressure on the health care system [13–15]. However, implementing NPIs carries societal and economic implications, particularly on already disadvantaged groups [16], and this needs to be taken into consideration by authorities. Despite this being highlighted as a global priority research area [17], there have been relatively few studies examining the impact of COVID-19 NPIs on people living in low- and middle-income countries [18–24].

At the time when this study began (May 2020), Thailand had implemented a number of NPIs, including nighttime curfews, restrictions on local movement, international travel and border closures, business closures and government guidance on social distancing, hand hygiene and mask-wearing [6, 25, 26] Details of these interventions and their effects on the transmission of COVID-19 have been published elsewhere [6]. In international comparison, Thailand during this time was deemed to have a relatively high government stringency index of between 75.0 to 52.8 (number ranging from 1 to 100, with 100 being the strictest) [27].

With many Thai people heavily impacted by NPIs–particularly informal or freelance workers, many of whom lost their jobs and income–the government introduced various social protection and financial support schemes in April 2020 to help its citizen cope [28]. These programmes included discounted electricity and water bills, low-interest loans and financial compensation for lost income (70% of current salary for 6 months for citizens covered under the social security scheme and 500 baht/USD160 per month for three months for those not covered) [28]. In a recent survey comparing the impact of NPIs across five countries during the first pandemic wave, Thai respondents reported being economically most negatively affected by these public health measures [29–31]. It is thus important to gain in-depth understanding of the impact and lived experiences of stringent public health measures during the COVID-19 pandemic—a research gap which our study seeks to address.

### Aims and scope

In this paper, we report findings from a qualitative study on how a small number of Thai residents viewed the COVID-19 public health measures introduced during the first wave of the pandemic in Thailand, the challenges they experienced during the first COVID-19 wave and lockdown, and their ways of coping with these challenges. Qualitative research methods aim to study a specific issue or phenomenon in a particular population, place, and context. Hence, while findings from qualitative research do not seek to be generalizable, they can provide in-depth and nuanced insights into the complexities of a particular locality, socio-cultural population, geographic or temporal context [32]. This understanding is important in order to guide policy makers to develop context-relevant responses that can minimize the negative impacts of NPIs on people’s lives amidst the ongoing COVID-19 pandemic.

We examine both practical and socio-culturally specific coping strategies. Practical coping strategies can be understood as problem focused coping strategies [33], which are employed by individuals to make physical adjustment to the performance of daily tasks, a particular situation or environment, to avoid or reduce negative outcomes and stressors. However, focusing solely on practical coping strategies, which emphasize the individual and the physical environment is likely to overlook more collectivist and culturally oriented conceptualisations of coping, which have been shown to be important predictors of resilient outcomes, such as among African-American populations [34, 35]. Scholars in cross-cultural psychology have thus argued that there is value in studying culturally and contextually informed paradigms of coping with stress and adversity [36]. In this paper, we therefore distinguish between practical coping (e.g., financial coping) and embedded socio-cultural coping (e.g. spiritual coping), in order to better identify coping strategies which are culturally specific and relevant to the Thai cultural context.

This qualitative study forms part of a larger mixed-methods study, entitled ‘Social, ethical and behavioural aspects of COVID-19’ (SEBCOV) conducted in Thailand, Malaysia, Italy, Slovenia and the UK [37]. The two strands of this study were a quantitative self-administered, anonymous online survey; and qualitative interviews with participants from Thailand, Malaysia, Italy and the UK (excluding Slovenia, where only the survey was conducted). The survey was launched on 1 May 2020, immediately following the implementation of strict NPIs among all participating countries, including quarantine, isolation, and social distancing. The survey included questions about the perceived economic and social impacts of NPIs, participants’ voluntary behavioural changes and self-reported compliance, and the prevalence of misinformation and ‘fake news’. The results of the cross-country [29, 30] and Thai survey study [31] have been published elsewhere and provide a quantitative overview of the socio-economic impact of the first pandemic wave, based on large respondent numbers from each country (5058 respondents in total, 1476 from Thailand). Results from the cross-country comparison qualitative SEBCOV study have also been published [37]. Complementary to those findings, this study provides an in-depth qualitative analysis into the unique perspectives and experiences of Thai residents during first wave of COVID-19 NPIs, specifically focusing on the Thai socio-cultural context.

Recent scientific advances on COVID-19 vaccinations and treatments have meant that lockdowns are largely being replaced with other strategies to mitigate the impact of SARS-CoV-2. However, in March 2022, both China and Samoa re-introduced lockdown measures to fight new outbreaks [38, 39]. In addition, as the prospect of new and emerging pandemics in the future is inevitable, particularly in light of the climate crisis, it is essential for pandemic preparedness planning to continue after the COVID-19 pandemic subsides [40, 41]. Findings from studies such the SEBCOV study can thus provide valuable insights for future decisions on implementing governmental restrictions and subsidization schemes.

## Methods

### Context

A total of 28 interviews were conducted between 8 May 2020 (approximately three months after the first documented COVID-19 case in Thailand) and 21 July 2020. This represents the period in which case numbers were steadily decreasing and government-imposed NPIs were being relaxed in Thailand. At the time of the interviews, all participants had already experienced the strictest lockdown imposed between 26 March and 3 May 2020, and the first wave of the pandemic (mid-March to early May 2020).

### Participant selection

Participants were recruited through existing personal and professional networks and selected using purposive sampling to gain a maximum variation sample, based on the following characteristics: age, gender, educational level, number of people living in the household, location, occupation, and risk of contracting COVID-19.

For this study, we defined high-risk groups as people living or working in contexts with high risk of COVID-19 infection, such as working in public places with exposure to a large number of people and unable to keep a distance of two meters [42]. Using these criteria, we defined hotel staff, tour guides, healthcare professionals, police officers, COVID-19 frontline healthcare staff, airport staff, hairdressers, masseurs, and taxi drivers as high-risk groups. Those defined as being at low-risks of contracting COVID-19 included students, teachers, office workers, retirees and people working from home. According to local ethics committee guidelines, participants received 200 Thai Baht (7 USD) compensation for their time. Due to the COVID-19 social distancing measures, all except one participant were interviewed via telephone. One interview was conducted face-to-face following easing of government restrictions.

### Theoretical framework, data collection and analysis

This study was qualitative in nature, employing a phenomenological approach to understand lived experience as narrated by the study participants [43]. The interview topic guide was developed in English by the core study team from participating countries of the SEBCOV qualitative study [44], based on the research aims of the mixed methods SEBCOV study [29] and then translated to Thai (see S1 Text). In-depth interviews were used to encourage participants to describe their own lived experiences of COVID-19 public health measures. All interviews were conducted by Thai qualitative researchers (BN, TP, SR) in their native Thai language. Participants were briefed about the aims and purpose of the study over the phone and written informed consent was obtained electronically, including permission to audio record interviews. Interviews lasted between 45–100 minutes (except for one 20-minute interview) and were conducted at a time convenient for participants. Thematic analysis using the Framework Method were employed to analyse and code data into themes and sub-themes [45].

Given the importance of interviewer reflexivity and positionality in qualitative research, where the researcher acts as the research instrument [46], the Thai research team engaged in self-reflexivity throughout the research encounter, in order ensure the quality of data collected. BN is a senior medical anthropologist with over 25 years’ experience working in qualitative research and acted as the lead for data collection and analysis in this study. TP is the SEBCOV project coordinator and has worked in qualitative research for over three years. SR is an early career researcher with a social science background and has received training in qualitative research. This variation in the interviewer characteristics among the Thai research team (e.g., gender, age, disciplinary background) supported the qualitative inquiry process during the study, both in terms of data collection (e.g., deciding which interviewer was most suited to interviewing each participant), as well as allowing for rich discussions of emerging findings among the research team. Prior to the data collection, BN provided data collection training for the team, including using role play with the topic guide, emphasizing in-depth interviewing skills, building rapport between interviewer and participants, and creating safe conversational spaces. Throughout this study, the Thai research team also engaged in reflective discussions with the UK-based cross-country qualitative research lead (MLS) and the SEBCOV qualitative teams in other countries [37], which supported methodological reflections, as well as the trustworthiness of interpretations and analysis.

Analysis was done iteratively, beginning as soon as the first interview was transcribed and continuing throughout the study in tandem with data collection. To ensure the quality of analysis, emerging findings from interviewers were regularly discussed among the Thai research team in order to arrive at a rich and trustworthy interpretation of results. De-identified transcripts were imported to NVivo software (v12) to organize and manage qualitative data. Three researchers (BN, TP, SR) performed coding independently to ensure the credibility and trustworthiness of the analysis (researcher triangulation) [47].

### Ethical approval

Ethical approval for this study was received from the Ethics Committee of the Faculty of Tropical Medicine, Mahidol University, Bangkok, Thailand (TMEC 20–016) and Oxford Tropical Research Ethics Committee (520–20).

## Results

Our results describe the views of COVID-19 public health measures, the challenges faced during the first COVID-19 wave and lockdown, and ways of coping with these challenges among a small number of Thai residents. The key themes arising from the analysis of interviews are discussed in turn below.

### Participant characteristics

A total of 28 Thai nationals, aged between 18 and 74 years participated in this study. Table 1 shows participants’ characteristics. Participants represented a range of occupational backgrounds, with 14% being healthcare workers and 86% coming from other occupations. Fifty-four percent of participants reported having completed tertiary education, 32% completed secondary and 14% had primary education. Forty-three percent of participants resided in Bangkok at the time of interview, with the remaining 57% residing across other, primarily rural regions of Thailand (see Table 1). The sample also included 17% of participants from non-binary gender due to Thailand’s large proportion of people who self-identify as gender diverse.

**Table 1:** Participant characteristics.

Participant characteristics	n (%)
**Gender**	n = 28
Female	12 (42.9)
Male	11 (39.3)
Other/non-binary	5 (17.9)
**Age range**	
18–24	2 (7.1)
25–34	5 (17.9)
35–44	12 (42.9)
45–54	4 (14.3)
55–64	3 (10.7)
65–74	2 (7.1)
**Highest level of education**	
Primary	4 (14.3)
Secondary	9 (32.1)
Tertiary	15 (53.6)
**Geographical region**	
Eastern	3 (10.7)
Northern	2 (7.1)
Southern	2 (7.1)
North-eastern	3 (10.7)
Central	6 (21.4)
Bangkok	12 (42.8)
**Number of household members**	
1	5 (17.9)
2	7 (25.0)
3	6 (21.4)
4	4 (14.3)
5	3 (10.7)
6	1 (3.6)
7	1 (3.6)
>7	1 (3.6)
**Occupation** ^**a**^	
1 Managers^b^	2 (7.1)
2 Professionals^c^	5 (17.9)
3 Technicians and Associate Professionals^d^	2 (7.1)
4 Clerical Support Workers^e^	7 (25.0)
5 Service and Sales Workers^f^	4 (14.3)
8 Plant and Machine Operators and Assemblers^h^	3 (10.7)
9 Elementary Occupations^i^	1 (3.6)
Others (not specified in ISCO-08)	4 (14.3)
University student	2 (7.1)
Retired	2 (7.1)
**Occupational category**	
Healthcare worker	4 (14.3)
Non-healthcare worker	24 (85.7)

^a^ Occupations have been classified according to the International Standard Classification of Occupations 08 (ISCO-08) [108].

### 1. Views of COVID-19 public health measures

Most participants expressed that they agreed with NPIs, saying that they believed the measures helped to control the spread of COVID-19 infections and deaths. While several participants stated this view as a reason for compliance with the measures, healthcare professional and healthcare workers were particularly vocal about supporting government’s key public health measures NPIs, seeing this as an extension of their professional duty:

*“I accept and follow these measures*. *I am sure that the government has thought through and researched the measures before recommending them*, *and that they will help control and protect us from getting COVID-19*.*”* (#4, female, 35 years, public health officer)

Similarly, several participants discussed feeling motivated to follow public health measures, not only out of a desire to protect themselves, but from a sense of solidarity towards and duty to protect their family, community, and country:

*“I follow the government’s recommendations strictly*. *First*, *I am 73 and I do not want to get sick and be a burden to others*. *One COVID-19 patient requires one doctor and three to four nurses to care for them*, *which could cost almost one million Baht to cure one patient*. *I am taking responsibility for myself and for the country*.*”* (#10, female, 71 years, retired teacher)

Some drew comparisons between the Thai government’s measures and those of other countries, feeling that the pandemic had been handled well in Thailand, due to both the government and the public working together “to take responsibility” (#2, male, 30 years, hotel bar manager) for controlling the spread of COVID-19:

*“You can see many countries are failing to control the spread [of COVID-19]*, *although they have better health care systems than Thailand*. *I absolutely agree with what the Thai government has done*. *It is not just the government’s duty to control the disease*. *Everyone needs to do their own bit*, *otherwise*, *we cannot overcome this critical time*.*”* (#9, female, 42 years, village health volunteer)

Some participants explicitly contrasted this description of solidaristic compliance with behaviours of non-compliance, which they described as ‘selfish’:

*“It is necessary that everyone follows these recommendations*. *It will help stop the spread*. *If we can stop the spread*, *we can go back to live normally*. *If we were selfish*, *we don’t keep distance*, *don’t wear masks*, *or we like to challenge the government*, *we would be suffering for a very long time*.*”* (#8, female, 40 years, nurse)

However, some participants also criticised the COVID-19 public health measures, with some feeling that the government had not thoroughly considered the ramifications of some of the measures imposed:

*“The government has authorized only one bank to issue the money for the 5000 Baht [financial support] scheme*. *Then all the people gathered at the bank*. *The government announced public holidays to make people stay home*, *but people rushed back to their hometown and gathered at the bus stations*. *These caused large gatherings and made it impossible to practice social distancing*.*”* (#10, female, 71 years, retired teacher)

Others voiced that certain restrictions did not make sense, with one participant describing her frustration about the guidance introduced around dining in restaurants following the first phase of relaxation of public health measures:

*“It doesn’t make sense that people from the same family have to sit separately in the restaurant*. *They live in the same house and go to the restaurant in the same car*. *But once they get into the restaurant*, *they are not be allowed to sit at the same table*.*”* (#9, female, 42 years, village health volunteer)

The lack of enforcement around implementing the Thai contact tracing app *Thai Chana* was also criticized by a few participants:

*“The app Thai Chana does not make sense*. *When we scan this app before entering a department store*, *staff don’t check whether we really checked-in on the app or not*.” (#24, non-binary gender, 24, tour guide)

### 2. Challenges experienced during first lockdown

Participants discussed the major challenges arising from the introduction of COVID-19 public health measures and from living through the first pandemic wave and lockdown more generally, including financial hardship; social isolation and loneliness; stigma and shaming; and fear of COVID-19 infection.

#### Financial challenges

Financial hardship during the lockdown was a key challenge discussed by many participants, most of whom reported having held paid jobs before the pandemic. For many, the introduction of public health measures resulted in partial or complete loss of earnings:

*“As many people work from home*, *I don’t have many customers*. *As a breadwinner*, *I cannot stop selling my food*. *I want to go home*, *but I cannot do it either as there is nothing to do in my hometown which means no income*.*”* (#21, female, 42 years, street food vendor)

Others described facing an increase in expenses because of complying with public health measures:

*“My salary wasn’t reduced*, *but I have higher expenses in terms of transport costs*. *I used to go to work by bus*. *But since COVID-19 happened*, *I have been using taxis to avoid the crowds on public transport*.*”* (#15, female, 42 years, office worker)

Particularly those who worked in tourism or related industries—Thailand’s largest employment sector—described being hardest hit by the implementation of public health measures:

*“I hardly have customers nowadays*. *I couldn’t send the same amount of money to my children*. *Some days I ate only one meal*. *Most days I ate instant noodles and eggs*. *I stopped drinking alcohol to save money*.*”* (#12, male, 45 years, taxi driver)

In comparison to those with stable employment, participants with the least financial and social protection, including those who relied on daily incomes and piece work reported facing the greatest financial difficulties. For example, the street food vendor (#21), masseuse (#16), tour guide (#24) and translator (#22) all reported struggling to generate sufficient income or completely losing their income during the first lockdown.

#### Social isolation and loneliness

Another challenge commonly discussed by participants were experiences of social isolation and loneliness in response to the introduction of social distancing measures. Due to travel restrictions, many participants described being unable to travel to visit friends and family, and discussed the negative emotional impacts of family gatherings and other social activities being cancelled due to COVID-19 restrictions:

*“When the government announced the lockdown*, *I stayed in my room alone for almost two months*. *I didn’t see my daughter*. *We only video called*. *I didn’t see anyone either*, *as the workplace was closed*. *It was quite lonely*. *When you are in your room alone*, *you lose motivation and energy to do things*. *I also felt paranoid… [that] I got COVID*.*”* (#16, female, 47 years, masseuse)

Parents often discussed feeling sad about the lack of physical contact between their children and their grandparents, while younger participants often regretted not being able to spend time with their friends in person.

#### Stigma and shaming

A few participants also discussed experiences of stigma and shaming during the first COVID-19 lockdown. For example, following the closure of Thai boxing stadiums as one of the first COVID-19 public health measures due to an outbreak cluster at a boxing stadium in March 2020, one participant, who worked as a professional boxer, described being stigmatized in his community, leaving him feeling lonely and isolated:

*“When people knew that I am a boxer*, *they reacted as if I am a disease*. *They didn’t want to sell food to me*, *because the first super-spreader event came from a boxing stadium*. *I have been bullied so much*. *I can’t tell people what to think*. *If they want to think and they want to feel scared*, *it is their own problem*. *There are so many boxers in Thailand*. *If all the boxers were to spread the disease*, *the government wouldn’t have let us walk around outside*. *However*, *it took me over a month to be able to walk out from my room*.*”* (#3, male, 24 years, Thai boxer)

Due to government recommendations, strict public health measures were also implemented in the workplace, with various strategies being used to improve compliance. Some of these strategies resulted in shaming of those who did not comply, subsequently resulting in stigma. One participant discussed such instances of stigmatization at the workplace, describing how the fear of stigma and shaming was being purposefully employed to make employees comply with COVID-19 social distancing measures:

*“At my workplace*, *we have very strict rules that we must follow otherwise we will be reviled*. *We have to keep distance in the canteen*, *lift*, *entrance*, *and everywhere in the office*. *If we don’t do it*, *there will be a photo from the CCTV emailed to all staff*… *if anyone breaks the rules*, *they will publish and send an email to everyone to punish you*. *But in the email*, *it doesn’t say who is who is who*, *but they just send the CCTV image*. *So*, *everyone knows what kind of mistake you have made*.*”* (#26, non-binary gender, 36 years, bank officer)

#### Fear of COVID-19 infection

Additionally, in discussing their lived experience and challenges of living through the first wave of the pandemic more generally, the overwhelming number of participants reported struggling with feelings of anxiety and fear of contracting COVID-19, or of friends and family becoming infected. This prompted many to “*often feel paranoid that I have COVID”* and to fear that they may infect their loved ones, especially older relatives:

*“[T]he most important thing is that my parents are old*, *and my father is bedridden*. *I am afraid that I may spread the disease to them*.*”* (#2, male, 30 years, hotel bar manager)

Particularly healthcare workers and others in frontline jobs expressed heightened fears of contracting and spreading COVID-19 to others, as a result of their heightened occupational risk of coming into contact with the virus:

*“I have young children at home*. *I don’t know when I will be infected and have no idea what to do if I would be infected*. *I am afraid of getting it*. *I can only follow the hygiene practice*.*”* (#9, female, 42 years, village health volunteer)

### 3. Coping strategies

Participants identified various coping strategies to help address the challenges faced as a result of COVID-19 and related public health measures. These included practical coping strategies to reduce the physical risks of COVID-19 infection, and mitigate the mental health, financial and social impact of public health measures, as well as embedded socio-cultural coping strategies to address attitudinal and psychological impacts of the pandemic.

#### Practical coping strategies

Among the practical coping strategies discussed by participants, strategies to cope with the physical risks of COVID-19 infection were widespread. These primarily included avoiding leaving the house and efforts to reduce personal risks of COVID-19 infection, such as wearing a mask, washing hands regularly and other hygiene measures:

*“I put on all my protection gear to protect myself and the others”* (#4, female, 35 years, public health officer)

Particularity in absence of sufficient PPE, such as among healthcare and other frontline workers, some spent significant time and effort trying to keep safe:

*“Once I reached home*, *I walked into the house from the back door*. *I took all my work clothes off*, *soaked them in disinfectant and washed them thoroughly myself*.*”* (#9, female, 42 years, village health volunteer)

Several participants also said that the risk of contracting COVID-19 had prompted them to actively look after their physical health, such as by consuming healthier food, taking food supplements, and doing exercise at home.

*“I have been taking more vitamins and food supplements during this COVID period*. *Firstly*, *because I was afraid that I will get it… I need to keep myself strong and healthy*.*”* (#24, non-binary gender, 24 years, tour guide)

*“I rent a room by myself*. *It is so stressful to just be inside the room*. *I do some exercise*. *I use a hula-hoop*. *It makes me sweaty and I feel better*. *I need to keep strong and fit*. *I heard that if you are weak*, *you can get COVID easily*.*”* (#16, female, 47 years, masseuse)

To reduce pandemic-related stress, many participants mentioned taking steps to care for their mental and emotional wellbeing. These included finding things to do at home to pass the time, such as cooking, spending time with household members, doing online shopping, and learning new skills. Several participants also said that they tried to refrain from regularly checking the COVID-19 related news, which they said helped manage their fears and anxiety levels:

*“When COVID-19 first came*, *I checked the news almost every hour to see how many people had been infected*. *I was so paranoid*, *and I wanted to know how close COVID-19 infections were*. *My colleague and I always discussed the numbers and government schemes to help people*. *After a month*, *I was so exhausted*. *I decided to not listen to the news during work hours as I already heard them through my colleagues*. *I only looked at the news summary when I got home*.*”* (#15, female, 42 years, office worker)

Furthermore, financial coping strategies, to mitigate the economic impact of the lockdown were commonly discussed, most commonly among those working in the informal sector. This included accessing government financial support, as well as finding innovative solutions to address financial hardships arising from COVID-19 public health measures. Those eligible to receive the governmental 5000 Baht financial support (‘*Ngeon yeaw ya’*) said the scheme offered short-term financial support, though several commented on the fund being insufficient to cover their needs.

To reduce the financial burden created by the loss of income opportunities, many said they tried to reduce their spending, such as on food and recreational costs, while also trying to find additional sources of income:

*“I can save my recreational costs of eating out with friends after work… Once we finished work*, *we don’t have social gatherings or parties*. *We just go home directly*. *I packed my leftovers to eat for lunch at work*, *that also saves money*.*”* (#2, male, 30 years, hotel bar manager)

Those who had lost their income completely, with no work opportunities in Bangkok, described returning to their rural hometowns during the lockdown to live with their parents or relatives, to work in agriculture and farming:

*“I was still okay during the first month of the closing of the boxing stadium*. *After that there were only expenses and no income*. *I decided to go back to my hometown to do farm work*. *We have eggs*, *rice*, *fish and vegetables*. *No need to always use money like when living in Bangkok*.*”* (#3, male, 24 years, Thai boxer)

A few participants described finding new business opportunities amidst the restrictions, including setting up online businesses:

*“I completely lost my job*, *as there are no more tourists coming*. *Now I am helping my mum and my sister to sell chili paste online*. *It is going very well*. *I am now doing my own brand*.*”* (#24, non-binary gender, 24 years, tour guide)

Finally, to cope with the lack of in-person contact, participants described shifting social interactions online, such as by using social media or online meeting platforms:

*“We have changed to meet online using video calls through Zoom*. *I don’t really like it*, *but it is better than not meeting anyone at all*. *I am trying to think positively*. *Normally*, *when we meet*, *we could touch*, *hug*, *and the conversations were more fruitful”* (#24, non-binary gender, 24 years, tour guide)

Additionally, many participants said that the creation of community-based support programs—which were described as an integral part of Thai cultural values of kindness and generosity (‘*Namjai’* [48])—helped maintain a sense of social support and connection during the lockdown. Participants described a wide range of grassroots support initiatives aimed at helping those in need, including aid packages from the Royal family and Buddhist temples and sharing pantries (‘*Tu pan suk’)* set up by individuals, private businesses, and community organizations across Thailand during the early stages of the pandemic:

*“Thailand is a society full of ‘Namjai’ [acts of kindness and generosity]*. *You can see many people giving food to others who have lost their jobs or have no money*. *Many ‘Tu pan suk’ [sharing pantries] have been popping up everywhere in Thailand to help others*.*”* (#21, female, 35 years, office worker)

Such initiatives were also discussed in light of ‘making merit’ (‘*Tham-bun’*) [49, 50] by helping others, which was seen to be an important value in Thai culture. Participants believed that giving and sharing was one of the best ways of making merit, and so perceived these actions as a key strategy of coping with the hardships of the pandemic:

*“Three years ago*, *many of my colleagues asked me to set up this ‘Tu pan suk’ [sharing pantry]*. *I thought this western idea wouldn’t work in Thailand*. *I thought who wants to give*, *and who will monitor and manage the pantry*? *But once COVID came*, *I decided to set up the first pantry at the side of the hospital so people who collected stuff from the pantry wouldn’t be seen by the public*, *as they may feel embarrassed*. *Now we have five pantries spread around the hospital*. *It fits Thai people*, *as it is a way of ‘Tham-bun’ [making merit]*.*”* (#18, female, 43 years, dentist)

#### Embedded socio-cultural coping strategies

Alongside the many practical coping strategies discussed, many participants also described some socio-culturally unique ways of coping with the challenges, stress, and uncertainty experienced in response to the COVID-19 pandemic and related public health measures. These ways of coping reflect unique religious and spiritual believes, practices and attitudes and have been summarized below.

Many participants described taking refuge in religious teachings and communities to cope with the stress and uncertainty related to the COVID-19 pandemic. For example, some described practicing meditating, praying and chanting, listening to the advice of religious leaders and following scripture for guidance:

*“I can’t go to temples as they are closed or cannot offer food to the monks in the morning*. *I have a Buddhist chanting book at home*. *I chant*, *pray and meditate*. *It helps me get some peace of mind*.*”* (#16, female, 47 years, masseuse)

*“We do what our prophet teaches us to do*, *and don’t do what he tells us not to do*.*”* (#13, male, 72 years, Retired/Muslim elder)

Several Buddhist participants said that the Buddhist teachings on accepting ‘impermanence’ offered them a helpful perspective from which to understand the sudden disruptions to their lives caused by the pandemic and related public health measures:

*“Being a dentist is normally a very high salary job*. *Many of my friends work in clinics*. *COVID-19 has impacted them quite a lot*. *Their income source is only from the clinic*. *So*, *it is about accepting and letting go*. *Like we understand the fact of Buddhist dharma more*, *that everything is impermanent–coming*, *staying*, *and disappearing*. *Life is impermanent*.*”* (#18, female, 43 years, dentist)

Others highlighted the importance of cultivating inner conditions such as acceptance and letting go of worries and stress caused by conditions in the outside world, beyond one’s control:

*“The first thing is accepting*. *The disease is happening*, *and accepting that there will be some changes*. *I am lucky that my job is secure*. *I don’t experience financial impact*, *but my job is very high risk*. *I am doing COVID screening before people enter the hospital*. *So*, *I accept my role and condition*.*”* (#4, female, 35 years, public health officer)

Regardless of culture, religion age, and sexual orientation, many participants mentioned the benefits of trying to practice ‘staying in the present’ to deal with the uncertainties and challenges arising from COVID-19. Participants felt it was helpful to focus their attention on the present moment rather than worry about the future, such as the uncertainties and worries relating to COVID-19:

“*COVID-19 is like a wake-up call for humankind*. *There is nothing permanent*. *There is COVID today*. *Someday*, *it will be gone*, *and a new thing will happen*. *Then it turns to normal*, *and we will get accustomed to it*. *There is nothing permanent*.*”* (#23, non-binary gender, 25 years, lead marketing manager)

Another way of coping frequently mentioned was through maintaining one’s roles and sense of duty by continuing to support others despite the significant impact and interruptions caused by COVID-19 restrictions. While this sentiment was also shared by lay people, one participant, who was a monk, expressed his deepened religious duty when described the importance of continuing to fulfil and maintain his responsibilities in everyday life amidst emergent changes:

*“We [monks] had been informed not to go out on alms rounds in the morning and to perform monk’s duties*. *In Thai culture*, *people feel happy*, *calm and at peace when they see the yellow robes*. *I feel for the community*. *In this hard time*, *apart from physical support they need spiritual support as well*. *I asked my assistants to pack food and necessary household items that were donated to us to be distributed to the community and to check people’s well-being*. *We continue to support the people in whatever way we can*.*”* (#17, male, 53 years, monk)

## Discussion

This study provides in-depth insight into the lived experiences and views of 28 Thai residents on the COVID-19 pandemic and the impact of public health measures implemented during the first COVID-19 wave in Thailand. Participants in our study predominantly held positive views about the government’s pandemic response. However, many reported experiencing significant challenges during the first lockdown, including financial and social challenges, fear, loneliness, and stigma. Coping strategies reflected participant’s efforts to address the challenges arising from NPIs and the pandemic more generally, including both practical ways of coping with everyday challenges, as well as socio-culturally unique and embedded ways of facing challenges.

### Views

Participants in this study generally expressed agreement with government-imposed public health measures. This confirms and explains the findings from our quantitative SEBCOV study [30, 31], and other studies that have reported widespread agreement with government-imposed COVID-19 NPIs and high levels of compliance with public health measures among Thai residents, including voluntary behaviour changes before official government regulations were implemented [30, 31, 51]. By offering an in-depth exploration of the views of public health measures, this study helps to shed light on the reasons and motivations for high self-reported compliance and agreement among a small number of Thai residents. These include participants sense of duty to protect their families, communities, and country–with an emphasis on solidaristic behaviour and collective responsibility–and their belief that NPIs were necessary to control viral spread. Our findings suggested that participants who self-identified as high-risk groups, including health professionals and those in high contact jobs (e.g., those working in service and tourism), reported higher compliance and agreement with NPIs. By contrast, participants who lived in provincial areas and had less contact with other people outside their home reported less compliance with the public health measures. These findings are consistent with the results of the quantitate part of the Thai SEBCOV study [31].

Thailand’s success at containing the spread of COVID-19 during the first wave has been attributed to good collaboration from the public in observing strict COVID-19 measures, as well as a robust public healthcare system and government socio-economic stimulus packages [26, 52]. However, some participants in this study also voiced criticisms of the government’s response, citing a perceived lack of enforcement of COVID-19 measures and insufficient financial support schemes. These views mirror findings by others showing that Thai government social support programs were inefficient in reaching key vulnerable populations during the first lockdown [28].

### Challenges

Participants in this study reported experiencing significant economic, social, and emotional challenges during the first COVID-19 wave. In international comparison, Thailand’s COVID-19 government stringency index during the first lockdown indicates relatively high stringency, with scores ranging between 52.8 to 75.0 (range: 1–100, with 100 indicating the strictest measures) [27]. Given Thailand’s heavy reliance on informal sector workers, which make up over half of those in the workforce, and on tourism, which accounted for 15% of GDP pre-pandemic [53], many participants unsurprisingly reported experiencing significant financial challenges, including reductions or loss of income and employment in response to NPIs. These findings echo results from the quantitative SEBCOV study, which showed financial challenge to be the foremost concern for Thai residents during the first COVID-19 wave, with respondents with lower levels of education most likely to report loss of earnings and loss of job [30]. Similarly, a study investigating the impact of COVID-19 lockdowns on street vendors in Vietnam, Thailand and Laos found that, in light of lacking governmental financial support, vendors experienced sharp drops in income and significant livelihood shocks in response to public health restrictions, further exacerbating income inequalities between vendors (e.g., those selling essential versus non-essential items) [54].

Findings from this study align with others who have argued that while the early pandemic response in Thailand was highly effective at tackling the physical risks of viral transmission, it fell short in addressing the socio-economic dimensions of the COVID-19 crisis, including inadequate provisions to low-income and vulnerable families and those affected most by NPIs, such as those working in the tourism sector [55]. In 2021, the Thai government launched initial COVID-19 social protection programmes to address people with multiple disadvantage, including a policy to support people with disability through fast track vaccination, moratorium on loan repayments, and extensions on applying to emergency loans without interest [56]. However, government support programs (financial and social) should be expanded to meet the needs of other populations vulnerable to experiencing the negative impacts of COVID-19 public health measures, including informal workers [54]; those on income from non-permanent or freelance contracts [13, 22]; younger and older people (18–24 years and 65+ years); those living in large households [30]; transnational migrants; and people living in informal settlements [57]. In the long-term, a focus on enhancing economic resilience such as by diversifying the economy, and providing support to grassroots and community-level networks is likely to benefit multiply disadvantaged populations in Thailand [55]. For these efforts to be equitable and effective in meeting the needs of those most vulnerable, the government needs to take a comprehensive public health approach, which accounts for the lived reality of multiply marginalized populations, and addresses social determinants of health, social protection mechanisms and health system preparedness [58].

Furthermore, participants in this study discussed prevalent socio-emotional challenges including fears and anxiety about COVID-19 infection in self and others, and isolation and loneliness due to prolonged social distancing. In line with these findings, other studies conducted in Thailand found that isolation due to lockdown and fears of COVID-19 infection were associated with higher rates of stress, depression, anxiety, panic, post-traumatic stress disorder, suicidal ideation, and suicide [6, 59]. A large survey conducted among the Thai general population during the first lockdown found that moderate levels of anxiety and little perceived control over infection risks were associated with non-evidence based behaviour changes like food stockpiling and consumption of Vitamin C supplements [60], mirroring findings from previous pandemics [61].

Participants also described experiences of isolation and loneliness as substantial challenges, which for many were closely linked to fears of infecting loved ones or of becoming infected themselves. Similarly, a study conducted among Thai Hill Tribe communities showed that interruption to social interactions and limited social contact among community members was regarded as a key negative impact of COVID-19 measures [62]. Close family and community ties and frequent social interactions are considered core elements of Thai culture and society, with these having been severely interrupted by social distancing guidelines in the early stages of the pandemic [63]. Together, these findings highlight the inadvertent psychological distress resulting from COVID-19 public health measures, which have received growing attention internationally [22, 64, 65] and in Thailand [59, 62, 66, 67]. Furthermore, these findings suggest the need for governments to place stronger emphasis on safeguarding mental health amidst this ongoing pandemic, the impacts of which are likely to outlive its physical health impacts.

Stigma, shaming, and discrimination of those suspected of having COVID-19 or of not adhering to public health measures was a further challenge that surfaced in this study. Despite being discussed by only a small number of participants, this finding appears significant in light of abundant research documenting the negative impact of stigma in the context of infectious disease prevention and control (e.g., SARS, HIV/AIDS, tuberculosis [68], and Ebola [69, 70]). Novel pathogen threats have been commonly shown to lead to stigmatisation and ostracization of particular population groups, in order to gain a false sense of security [71, 72]. Healthcare workers worldwide have reportedly been subjected to heavy stigmatisation and discrimination during this pandemic [73, 74], including in Thailand [75, 76]. Similarly, in this study, one participant who belonged to an occupational group in which an outbreak cluster occurred during the early stages of the pandemic (professional boxing), reported stigmatisation by community members. Another participant described the use of social shaming and stigma to incite compliance with social distancing measures among employees in the workplace. Particularly in the early stages of the COVID-19 pandemic in Thailand, fear of the virus, ambiguous information and social isolation and quarantining practices are likely to have heightened social stigma, blame and ‘othering’ [77]. Additionally, being a collectivist-oriented rather than self-oriented society, Thai culture places strong emphasis on group belonging, family ties, and clan protection in exchange for loyalty [78]. Transgression of cultural norms are thus likely to result in substantial stigma, shaming and social ostracism [78], which may help explain our findings.

Together, our findings on COVID-19 related stigma and discrimination not only point towards the psychological and physical risks facing individuals subjected to stigmatization, but also point towards the harms of stigma for society as a whole, resulting from potential concealment of infections due to fear of reprisal; delayed access to testing and treatment; underreporting and ongoing transmission of COVID-19 [79]. The World Health Organization has published guidance to address social stigma associated with COVID-19, such as the use of inclusive language and less stigmatizing terminology, highlighting that ‘words matter’ [80]. This includes avoiding references to place of origin of the virus and not referring to those infected with COVID-19 and spreading the virus as ‘super spreaders’, such as seen in Thailand surrounding professional boxers. Others have stressed the importance of taking a rights-based approach within public health programmes to counteract and prevent COVID-19 related stigma and discrimination in the context of LMICs [81]. In an initial attempt to mitigate COVID-19 related stigma, the Thai Centre for Covid-19 Situation Administration (CCSA) spokesperson issued a statement on 9 May 2021 condemning the stigmatisation of certain workplaces and their staff related to COVID-19 transmissions [82]. Despite these efforts, there is a need for further public engagement, health communication and information campaigns in Thailand to take effective action to counteract COVID-19 related stigma and ‘othering’ in the media, workplaces, and society more broadly in order to facilitate infection control [83].

Importantly, research from across the world has shown that the burdens of COVID-19 and related public health measures are shouldered unequally across society, with those from lower-income countries, lower socio-economic status and poorer households being most negatively impacted [16, 30, 84, 85]. Similarly, findings from this study and the quantitative SEBCOV study [30, 31], indicate important differences in the extent of psycho-social and financial challenges experienced by participants. Interview participants working in the informal economy and on the ‘frontlines’, including healthcare workers, reported highest levels of fear of COVID-19 and high work-related impacts of public health measures (e.g., through loss of earnings and employment, or higher risk of exposure due to inability to work from home), and similar impacts were reported by our survey participants (e.g. in Thailand, 91% of those on contract and freelance incomes reported loss of earnings, and 23% reported loss of job; [20]). This also echoes other research from Thailand showing differences in the levels of COVID-19 related fear by geographic region and population group [61]. Therefore, to prevent further entrenchment of existing health, economic and social inequalities caused by COVID-19 and related public health measures, Thai policy makers need to prioritise supporting socially and economically vulnerable populations, including individuals from poor households, and those working in the tourism industry and the informal economy.

### Coping

Participants identified numerous practical strategies for coping with the negative impacts of NPIs and the pandemic more broadly. Aside from strategies to reduce personal risks of COVID-19 infection, through social distancing and PPE use, participants reported taking active steps to care for their mental and emotional wellbeing and reduce the economic impacts of lockdown. These included finding new income sources or moving back to their hometowns to reduce spending or to find temporary work in farming.

Moreover, many participants described unique and socio-culturally embedded strategies of coping with the challenges, stress, and uncertainty of NPIs and the pandemic more broadly, most notably through recourse to spirituality and religion. The religious and spiritual beliefs, attitudes, and practices discussed in our study included engaging with prayer and meditation; making merit (‘*Tham-bun*’) through kindness, generosity and sharing (‘*Namjai’*); and following Buddhist virtues of acceptance and ‘letting be’. These socio-culturally embedded beliefs and practices played a significant role in participants’ attempts to cope with COVID-19, making them feel more resilient and at peace in the face of uncertainty and loss. Mills and colleagues describe the Thai culturally embedded coping strategy of acceptance *(‘Thum-jai’)* as “accepting and letting go of the negative situation, forgetting the bad feeling, calming or steadying the mind, and developing patience and understanding” [86], finding that the emotion-based coping strategy of *‘Thum-jai’* helped Thai people develop purposeful approaches of thinking and acting in light of adverse events which cannot be changed [87]. Our findings also echo other research on the positive association between social support and coping from Thailand (e.g., HIV [88], breast cancer [89, 90] and COVID-19 [91]) and internationally (e.g., religious coping in the context of COVID-19 [92–95], healthy ageing [96] and terminal illness [97]), highlighting the important and beneficial role of spirituality and religion in coping with adverse health events and crises. Similarly, a qualitative study examining mental health impacts and coping strategies among disadvantaged groups in India during COVID-19 lockdown found that religion and devotional practices played an important role in participants coping strategies, by helping them make sense and find meaning in the COVID-19 crisis. As such, participants described coping by accepting and “knowing all events are in the hands of *Uparwala* (the One Above)” [18].

Findings from this study provide a rich understanding of the unique ways in which Thai people coped during the early stages of the pandemic, which can help support the designing of culturally sensitive COVID-19 measures in future. While large in-person religious gatherings have significant potential to contribute to COVID-19 transmission [98, 99], governments should be mindful of the important role of religious and socio-cultural practices in coping and resilience, and make efforts to establish infrastructure and environments that facilitate safe engagement with religious practice amidst this pandemic and in future public health emergencies (e.g., online platforms for prayer, teaching, and religious community gatherings). Additionally, drawing upon existing religious values and community structures, and working with spiritual and religious leaders to inform public health interventions, communication and information campaigns may help support a greater sense of social cohesion and wellbeing [100].

### Strengths and limitations

This study has several strengths and limitations. To our knowledge, it is the first qualitative study to investigate lived experiences among the Thai public during the first wave of the COVID-19 pandemic. It also provides in-depth insights that complement findings from the quantitative online survey conducted in Thailand and other countries as part of the larger SEBCOV mixed-methods study [30, 31]. Furthermore, using purposive sampling, we were able to recruit a number of participants belonging to groups disproportionately affected by COVID-19, including older people and gender minority populations [101], healthcare workers, and those working in the informal sector and in close contact jobs [102]. However, because of recruitment being conducted through existing personal and professional networks and requiring digital literacy skills, there was an overrepresentation of participants from tertiary education levels (53%) within our sample (vs. 21% of Thai people over 25 years who completed at least short-cycle tertiary education) [103]. Our findings are thus unable to reflect the views, challenges, and coping strategies of multiply marginalized populations, such as those with lower (digital) literacy and educational levels, suggesting that our findings underrepresent the voices of those most marginalized by the COVID-19 public health measures.

Our sample also included a large proportion of participants from non-binary gender (17). Despite gender diversity being more visible and socially tolerated in Thailand than in many other societies, gender diverse populations are likely to experience greater mental health challenges and economic instability during the COVID-19 pandemic due to facing multiple disadvantages [104, 105]. The inclusion of this commonly underrepresented group is thus important to better understand the needs of this multiply marginalized population.

All interviews (except one) and recruitment were conducted remotely, which presents both challenges and opportunities [106]. In our study, remote data collection enabled nationwide recruitment of interview participants, but also presented limitations, including the potential exclusion of participants who were illiterate, did not have smartphones or lacked internet access. Furthermore, while some participants may have felt more comfortable conducting remote rather than in-person interviews, thereby facilitating open dialogue, for others, the reduced opportunities to build trust and rapport remotely may have resulted in a lack of trust and openness during the phone interview [106]. During one interview, we encountered challenges to build sufficient rapport with the participant remotely, which resulted in a short 20-minute interview and a lack of richness of data. Additionally, some participants may have underreported their challenges and frustrations or overreported adherence and agreement with the measures, due to fears of reprisal for expressing dissenting opinions and criticizing the government’s response [107].

## Conclusion

This study highlights key economic and psychosocial challenges faced by a small number of Thai residents in response to the public health measures implemented during the first wave of the COVID-19 pandemic in Thailand. Socio-culturally embedded coping strategies played a central role in managing pandemic stress and lockdown related challenges, suggesting the importance of considering socio-cultural context when designing and implementing interventions to support community resilience in times of crisis. Findings from this study remain largely relevant in light of low COVID-19 vaccine availability and ongoing public health restrictions in Thailand and underscore the need for continued political efforts to help mitigate the negative impacts of NPIs on the public. In particular, government interventions are needed to support income security, mental health and coping, especially for vulnerable and marginalised populations.

## Supporting information

S1 Text Topic guide. (DOCX)

## References

[1] World Bank. World Bank Country and Lending Groups 2021 [10.10.2021]. Available from: https://datahelpdesk.worldbank.org/knowledgebase/articles/906519-world-bank-country-and-lending-groups.

[2] Britannica. Thailand 2021 [10.10.2021]. Available from: https://www.britannica.com/place/Thailand.

[3] Statista. Thailand: Share of economic sectors in the gross domestic product (GDP) from 2010 to 2020 2022 [12.01.2022]. Available from: https://www.statista.com/statistics/331893/share-of-economic-sectors-in-the-gdp-in-thailand/.

[4] Department of Disease Control (Thailand). Corona Virus Disease (COVID-19) by Department of Disease Control 2020 [14.04.2021]. Available from: https://ddc.moph.go.th/viralpneumonia/eng/index.php.

[5] World Health Organization. Thailand COVID-19 situation report 1st May 2020 2020 [15.07.2021]. Available from: https://www.who.int/docs/default-source/searo/thailand/2020-05-01-tha-sitrep-69-covid19-th.pdf?sfvrsn=11dff472_2.

[6] Triukose S, Nitinawarat S, Satian P, Somboonsavatdee A, Chotikarn P, Thammasanya T, et al. Effects of public health interventions on the epidemiological spread during the first wave of the COVID-19 outbreak in Thailand. PLoS One. 2021;16(2):e0246274. doi: 10.1371/journal.pone.0246274 33606734 PMC7894963

[7] Rajatanavin N, Tuangratananon T, Suphanchaimat R, Tangcharoensathien V. Responding to the COVID-19 second wave in Thailand by diversifying and adapting lessons from the first wave. BMJ Global Health. 2021;6(7):e006178. doi: 10.1136/bmjgh-2021-006178 34285042 PMC8295022

[8] World Health Organization. COVID-19—WHO Thailand Situation Reports 2021 [01.12.2021]. Available from: https://www.who.int/thailand/emergencies/novel-coronavirus-2019/situation-reports.

[9] Tourist Authority of Thailand Newsroom. Thailand launches ‘Thai Chana’ online platform to retain effectiveness in COVID-19 control measure 2020 [20.12.2020]. Available from: https://www.tatnews.org.

[10] Our World in Data. Thailand: Coronavirus Pandemic Country Profile 2021. Available from: https://ourworldindata.org/coronavirus/country/thailand.

[11] World Health Organization. Non-pharmaceutical public health measures for mitigating the risk and impact of epidemic and pandemic influenza 2019 [13.04.2021]. Available from: https://www.who.int/influenza/publications/public_health_measures/publication/en/. 42118904

[12] Prevention CfDCa. Non-pharmaceutical interventions (NPIs) 2020 [13.04.2021]. Available from: https://www.cdc.gov/nonpharmaceutical-interventions/index.html.

[13] Teslya A, Pham TM, Godijk NG, Kretzschmar ME, Bootsma MC, Rozhnova G. Impact of self-imposed prevention measures and short-term government-imposed social distancing on mitigating and delaying a COVID-19 epidemic: A modelling study. PLoS medicine. 2020;17(7):e1003166. doi: 10.1371/journal.pmed.1003166 32692736 PMC7373263

[14] Doung-Ngern P, Suphanchaimat R, Panjangampatthana A, Janekrongtham C, Ruampoom D, Daochaeng N, et al. Case-control study of use of personal protective measures and risk for SARS-CoV 2 infection, Thailand. Emerging infectious diseases. 2020;26(11):2607. doi: 10.3201/eid2611.203003 32931726 PMC7588529

[15] Koo JR, Cook AR, Park M, Sun Y, Sun H, Lim JT, et al. Interventions to mitigate early spread of SARS-CoV-2 in Singapore: a modelling study. The Lancet Infectious Diseases. 2020. doi: 10.1016/S1473-3099(20)30162-6 32213332 PMC7158571

[16] Bottan N, Hoffmann B, Vera-Cossio D. The unequal impact of the coronavirus pandemic: Evidence from seventeen developing countries. PLoS One. 2020;15(10):e0239797. doi: 10.1371/journal.pone.0239797 33027272 PMC7540865

[17] Norton A, Gozalo ADLH, De Colombi NF, Alobo M, Asego JM, Al-Rawni Z, et al. The remaining unknowns: a mixed methods study of the current and global health research priorities for COVID-19. BMJ Global Health. 2020;5(7):e003306. doi: 10.1136/bmjgh-2020-003306 32727843 PMC7431769

[18] Mathias K, Rawat M, Philip S, Grills N. “We’ve got through hard times before”: acute mental distress and coping among disadvantaged groups during COVID-19 lockdown in North India-a qualitative study. International journal for equity in health. 2020;19(1):1–12. doi: 10.1186/s12939-020-01345-7 33334344 PMC7745174

[19] Hamadani JD, Hasan MI, Baldi AJ, Hossain SJ, Shiraji S, Bhuiyan MSA, et al. Immediate impact of stay-at-home orders to control COVID-19 transmission on socioeconomic conditions, food insecurity, mental health, and intimate partner violence in Bangladeshi women and their families: an interrupted time series. The Lancet Global Health. 2020;8(11):e1380–e9. doi: 10.1016/S2214-109X(20)30366-1 32857955 PMC7447230

[20] Chackalackal DJ, Al-Aghbari AAA, Jang SY, Ramirez TR, Vincent J, Joshi A, et al. The Covid-19 pandemic in low-and middle-income countries, who carries the burden? review of mass media and publications from six countries. Pathogens and global health. 2021;115(3):178–87. doi: 10.1080/20477724.2021.1878446 33657984 PMC8079077

[21] Barkur G, Vibha GBK. Sentiment analysis of nationwide lockdown due to COVID 19 outbreak: Evidence from India. Asian journal of psychiatry. 2020;51:102089. doi: 10.1016/j.ajp.2020.102089 32305035 PMC7152888

[22] Zainudeen ZT, Abd Hamid IJ, Azizuddin MNA, Bakar FFA, Sany S, Zolkepli IA, et al. Psychosocial impact of COVID-19 pandemic on Malaysian families: a cross-sectional study. BMJ open. 2021;11(8):e050523. doi: 10.1136/bmjopen-2021-050523 34380732 PMC8359868

[23] Bahar Moni AS, Abdullah S, Bin Abdullah MFIL, Kabir MS, Alif SM, Sultana F, et al. Psychological distress, fear and coping among Malaysians during the COVID-19 pandemic. PLoS One. 2021;16(9):e0257304. doi: 10.1371/journal.pone.0257304 34506576 PMC8432783

[24] Norton A, Bucher A, Antonio E, Advani N, Grund H, Mburu S, et al. A living mapping review for COVID-19 funded research projects: three-month update. Wellcome Open Research. 2020;5. 10.12688/wellcomeopenres.16259.1 PMC7579366 33117894

[25] Ministry of Public Health, Thailand. Thailand’s experience in the COVID-19 response. 2020. [Available from: https://ddc.moph.go.th/viralpneumonia/eng/file/pub_doc/LDoc9.pdf accessed 17 April 2021].

[26] Socio-Economic Impact Assessment of COVID-19 in Thailand. October 2020. Available at https://www.th.undp.org/content/thailand/en/home/library/socio-economic-impact-assessment-of-covid-19-in-thailand.html. Accessed 14 May 2021.

[27] Hale T., Webster S. Petherick A., et al. Oxford COVID-19 Government Response Tracker, Blavatnik School of Government. 2020. Available from: https://covidtracker.bsg.ox.ac.uk/]. Accessed 14 May 2021. 10.1038/s41562-021-01079-8 33686204

[28] Pongutta S, Kantamaturapoj K, Phakdeesettakun K, Phonsuk P. The social impact of the COVID-19 outbreak on urban slums and the response of civil society organisations: A case study in Bangkok, Thailand. Heliyon. 2021:e07161. doi: 10.1016/j.heliyon.2021.e07161 34136704 PMC8180617

[29] Pan-Ngum W, Poomchaichote T, Cuman G, Cheah PK, Waithira N, Mukaka M, et al. Social, ethical and behavioural aspects of COVID-19. Wellcome Open Res. 2020;5:90. Epub 2020/07/28. doi: 10.12688/wellcomeopenres.15813.2 ; PubMed Central PMCID: PMC7355217. 32704548 PMC7355217

[30] Osterrieder A, Cuman G, Pan-Ngum W, Cheah PK, Cheah P-K, Peerawaranun P, et al. Economic and social impacts of COVID-19 and public health measures: results from an anonymous online survey in Thailand, Malaysia, the UK, Italy and Slovenia. BMJ open. 2021;11(7):e046863. doi: 10.1136/bmjopen-2020-046863 34285007 PMC8295020

[31] Pan-ngum W, Poomchaichote T, Peerawaranun P et al. Perspectives on public health interventions in the management of the COVID-19 pandemic in Thailand [version 2; peer review: 1 approved]. Wellcome Open Res 2020, 5:245 (10.12688/wellcomeopenres.16293.2). PMC8283548 34345714

[32] Leung L. Validity, reliability, and generalizability in qualitative research. Journal of family medicine and primary care. 2015;4(3):324. doi: 10.4103/2249-4863.161306 26288766 PMC4535087

[33] Folkman S, Lazarus RS. Manual for the ways of coping questionnaire: Consulting Psychologists Press; 1988.

[34] Mercier CM, Abbott DM, Ternes MS. Coping Matters: An Examination of Coping among Black Americans During COVID-19. The Counseling Psychologist. 2022:00110000211069598.

[35] Utsey SO, Bolden MA, Lanier Y, Williams O III. Examining the role of culture-specific coping as a predictor of resilient outcomes in African Americans from high-risk urban communities. Journal of Black Psychology. 2007;33(1):75–93.

[36] Kuo BC. Culture’s consequences on coping: Theories, evidences, and dimensionalities. Journal of Cross-Cultural Psychology. 2011;42(6):1084–100.

[37] Schneiders ML, Naemiratch B, Cheah PK, Cuman G, Poomchaichote T, Ruangkajorn S, et al. The impact of COVID-19 non-pharmaceutical interventions on the lived experiences of people living in Thailand, Malaysia, Italy and the United Kingdom: A cross-country qualitative study. PLoS One. 2022;17(1):e0262421. doi: 10.1371/journal.pone.0262421 35061789 PMC8782407

[38] CNN. In China, 37 million people are in Covid lockdown. Here’s what we know 2022. Available from: https://edition.cnn.com/2022/03/15/china/china-covid-outbreak-explainer-intl-hnk/index.html.

[39] RNZ. Covid-19 cases in Samoa increasing rapidly 2022. Available from: https://www.rnz.co.nz/international/pacific-news/463847/covid-19-cases-in-samoa-increasing-rapidly.

[40] Control ECfDPa. Why is pandemic preparedness planning important? 2022. Available from: https://www.ecdc.europa.eu/en/seasonal-influenza/preparedness/why-pandemic-preparedness

[41] Nature. Has COVID taught us anything about pandemic preparedness? 2021. Available from: https://www.nature.com/articles/d41586-021-02217-y. 10.1038/d41586-021-02217-y 34389832

[42] Qureshi Z, Jones N, Temple R, Larwood JPJ, Greenhalgh T, Bourouiba D. (2020). What is the evidence to support the 2-metre social distancing rule to reduce COVID-19 transmission?. Retrieved 20 December, 2020, from https://www.cebm.net/covid-19/what-is-the-evidence-to-support-the-2-metre-social-distancing-rule-to-reduce-covid-19-transmission/)

[43] Neubauer BE, Witkop CT, Varpio L. How phenomenology can help us learn from the experiences of others. Perspect Med Educ. 2019;8(2):90–7. Epub 2019/04/07. doi: 10.1007/s40037-019-0509-2 ; PubMed Central PMCID: PMC6468135. 30953335 PMC6468135

[44] Poomchaichote T, Naemiratch B, Schneiders M et al. 2020. Topic guide for qualitative study: Social, ethical and behavioural aspects of COVID-19 (Version Version 1.0, 30 April 2020). Zenodo. 10.5281/zenodo.377793. PMC7355217 32704548

[45] Gale NK, Heath G, Cameron E, Rashid S, Redwood S. Using the framework method for the analysis of qualitative data in multi-disciplinary health research. BMC Med Res Methodol. 2013;13:117. Epub 2013/09/21. doi: 10.1186/1471-2288-13-117 ; PubMed Central PMCID: PMC3848812. 24047204 PMC3848812

[46] Pezalla AE, Pettigrew J, Miller-Day M. Researching the researcher-as-instrument: An exercise in interviewer self-reflexivity. Qualitative research. 2012;12(2):165–85. doi: 10.1177/1487941111422107 26294895 PMC4539962

[47] Lincoln Y, Guba EG (1985). Naturalistic inquiry. Newbury Park, CA, Sage.

[48] Thai Royal Academy Dictionary 2011) (Homepage: Thai Royal Academy Dictionary https://dictionary.orst.go.th, Accessed 20 December 2020.

[49] Mulder JAN. Merit An investigation of the motivational qualities of the Buddhist concept of merit in Thailand. Social Compass. 1969;16(1):109–20.

[50] encyclopedia.com. Thai Religion 2021 [14.12.2021]. Available from: https://www.encyclopedia.com/environment/encyclopedias-almanacs-transcripts-and-maps/thai-religion.

[51] Maude RR, Jongdeepaisal M, Skuntaniyom S, Muntajit T, Blacksell SD, Khuenpetch W, et al. Improving knowledge, attitudes and practice to prevent COVID-19 transmission in healthcare workers and the public in Thailand. BMC Public Health. 2021;21(1):749. Epub 2021/04/19. doi: 10.1186/s12889-021-10768-y ; PubMed Central PMCID: PMC8053080. 33865342 PMC8053080

[52] Issac A, Radhakrishnan RV, Vijay VR, Stephen S, Krishnan N, Jacob J, et al. An examination of Thailand’s health care system and strategies during the management of the COVID-19 pandemic. J Glob Health. 2021;11:03002. Epub 2021/03/02. doi: 10.7189/jogh.11.03002 ; PubMed Central PMCID: PMC7897427 form at (available upon request from the corresponding author), and declare no conflicts of interest. 33643614 PMC7897427

[53] World Bank Group. Thailand Economic Monitor: Thailand in the Time of COVID-19 (English). 2020. [Available from: http://documents.worldbank.org/curated/en/456171593190431246/Thailand-Economic-Monitor-Thailand-in-the-Time-of-COVID-19 accessed 17 April 2021].

[54] Turner S, Langill JC, Nguyen BN. The utterly unforeseen livelihood shock: COVID‐19 and street vendor coping mechanisms in Hanoi, Chiang Mai and Luang Prabang. Singapore Journal of Tropical Geography. 2021;42(3):484–504. doi: 10.1111/sjtg.12396 34908627 PMC8661646

[55] Marome W, Shaw R. covid-19 response in Thailand and its implications on future preparedness. International journal of environmental research and public health. 2021;18(3):1089. doi: 10.3390/ijerph18031089 33530526 PMC7908435

[56] Department of Empowement of Persons with Disabilities. ข่าวประชาสัมพันธ์ 2022. Available from: https://dep.go.th/th/news/news-release.

[57] Schmidt-Sane M, Ripoll S, Wilkinson A. Key considerations for COVID-19 management in marginalised populations in Southeast Asia: Transnational migrants, informal workers, and people living in informal settlements 2020. Available from: https://opendocs.ids.ac.uk/opendocs/handle/20.500.12413/15324.

[58] Prasad V, Sri BS, Gaitonde R. Bridging a false dichotomy in the COVID-19 response: a public health approach to the ‘lockdown’debate. BMJ Global Health. 2020;5(6):e002909. doi: 10.1136/bmjgh-2020-002909 32527850 PMC7292046

[59] Bliźniewska-Kowalska KM, Halaris A, Wang S-C, Su K-P, Maes M, Berk M, et al. A Review of the Global Impact of the COVID-19 Pandemic on Public Mental Health, with a Comparison Between the USA, Australia, and Poland with Taiwan and Thailand. Medical science monitor: international medical journal of experimental and clinical research. 2021;27:e932220–1. 33972496 10.12659/MSM.932220 PMC8122850

[60] Goodwin R, Wiwattanapantuwong J, Tuicomepee A, Suttiwan P, Watakakosol R, Ben-Ezra M. Anxiety, perceived control and pandemic behaviour in Thailand during COVID-19: Results from a national survey. Journal of Psychiatric Research. 2021;135:212–7. doi: 10.1016/j.jpsychires.2021.01.025 33497875 PMC7826082

[61] Rubin GJ, Amlôt R, Page L, Wessely S. Public perceptions, anxiety, and behaviour change in relation to the swine flu outbreak: cross sectional telephone survey. BMJ. 2009;339. 10.1136/bmj.b2651 PMC2714687 19574308

[62] Kitchanapaibul S, Udplong A, Apidechkul T, Tamornpark R, Mulikaburt T, Srichan P, et al. Experiences and expectations regarding COVID-19 prevention and control measures among the hill tribe population of northern Thailand: a qualitative study. BMC Public Health. 2021;21(1):1–12. 34088306 10.1186/s12889-021-11145-5 PMC8176874

[63] Ministry of Public Health. Social distance guideline in Thailand Thailand2020 [10.10.2021]. Available from: https://ddc.moph.go.th/viralpneumonia/eng/file/news/news_no128_010663.pdf.

[64] Holmes EA, O’Connor RC, Perry VH, Tracey I, Wessely S, Arseneault L, et al. Multidisciplinary research priorities for the COVID-19 pandemic: a call for action for mental health science. The Lancet Psychiatry. 2020;7(6):547–60. doi: 10.1016/S2215-0366(20)30168-1 32304649 PMC7159850

[65] Rahman MA, Islam SMS, Tungpunkom P, Sultana F, Alif SM, Banik B, et al. COVID-19: Factors associated with psychological distress, fear, and coping strategies among community members across 17 countries. Globalization and health. 2021;17(1):1–19. 34598720 10.1186/s12992-021-00768-3 PMC8485312

[66] Pothisiri W, Vicerra PMM. Psychological distress during COVID-19 pandemic in low-income and middle-income countries: a cross-sectional study of older persons in Thailand. BMJ open. 2021;11(4):e047650. doi: 10.1136/bmjopen-2020-047650 33931412 PMC8098281

[67] Srichannil C. The COVID-19 pandemic and Thailand: A psychologist’s viewpoint. Psychological Trauma: Theory, Research, Practice, and Policy. 2020;12(5):485. doi: 10.1037/tra0000808 32478554

[68] Mak W, Cheung F, Woo J, Lee D, Li P, Chan K, et al. A comparative study of the stigma associated with infectious diseases (SARS, AIDS, TB). Hong Kong Med J. 2009;15:34–7. 20393211

[69] Davtyan M, Brown B, Folayan MO. Addressing Ebola-related stigma: lessons learned from HIV/AIDS. Global health action. 2014;7(1):26058. doi: 10.3402/gha.v7.26058 25382685 PMC4225220

[70] Overholt L, Wohl DA, Fischer WA, Westreich D, Tozay S, Reeves E, et al. Stigma and Ebola survivorship in Liberia: results from a longitudinal cohort study. PLoS One. 2018;13(11):e0206595. doi: 10.1371/journal.pone.0206595 30485311 PMC6261413

[71] Park JH, Schaller M, Crandall CS. Pathogen-avoidance mechanisms and the stigmatization of obese people. Evolution and Human Behavior. 2007;28(6):410–4.

[72] Faulkner J, Schaller M, Park JH, Duncan LA. Evolved disease-avoidance mechanisms and contemporary xenophobic attitudes. Group Processes & Intergroup Relations. 2004;7(4):333–53.

[73] Bagcchi S. Stigma during the COVID-19 pandemic. The Lancet Infectious Diseases. 2020;20(7):782. doi: 10.1016/S1473-3099(20)30498-9 32592670 PMC7314449

[74] Abdelhafiz AS, Alorabi M. Social stigma: the hidden threat of COVID-19. Frontiers in public health. 2020;8. doi: 10.3389/fpubh.2020.00429 32984238 PMC7484807

[75] Gunawan J, Juthamanee S, Aungsuroch Y. Current mental health issues in the era of Covid-19. Asian Journal of Psychiatry. 2020;51:102103. doi: 10.1016/j.ajp.2020.102103 32361675 PMC7182519

[76] Gunawan J, Aungsuroch Y, Marzilli C. ‘New Normal’in Covid-19 Era: A Nursing Perspective From Thailand. Journal of the American Medical Directors Association. 2020;21(10):1514. doi: 10.1016/j.jamda.2020.07.021 32859516 PMC7375267

[77] Thanirat สภก, Radabutr เ, Wichianrat สจร, Opasawatchai ศรพ Strategies to Relieve a Social Stigma on COVID-19. Kuakarun Journal of Nursing. 2020;27(2):164–74.

[78] Hofstede G. Dimensionalizing cultures: The Hofstede model in context. Online readings in psychology and culture. 2011;2(1):2307–0919.1014.

[79] Yuan K, Huang X-L, Yan W, Zhang Y-X, Gong Y-M, Su S-Z, et al. A systematic review and meta-analysis on the prevalence of stigma in infectious diseases, including COVID-19: a call to action. Molecular psychiatry. 2021:1–15. 34580416 10.1038/s41380-021-01295-8 PMC8475479

[80] IFRC WHOU. Social Stigma associated with COVID-19 2020. Available from: https://www.who.int/docs/default-source/coronaviruse/covid19-stigma-guide.pdf.

[81] Roelen K, Ackley C, Boyce P, Farina N, Ripoll S. COVID-19 in LMICs: the need to place stigma front and centre to its response. The European Journal of Development Research. 2020;32(5):1592–612. doi: 10.1057/s41287-020-00316-6 33100598 PMC7575856

[82] COVID-19) TCfC-SAศนขอล. อย่ารังเกียจ อย่าตีตรา: Government Website via Facebook; 2021. Available from: https://www.facebook.com/informationcovid19/posts/321757432775921/.

[83] Fischer LS, Mansergh G, Lynch J, Santibanez S. Addressing disease-related stigma during infectious disease outbreaks. Disaster medicine and public health preparedness. 2019;13(5–6):989–94. doi: 10.1017/dmp.2018.157 31156079 PMC6889068

[84] Lewnard JA, Lo NC. Scientific and ethical basis for social-distancing interventions against COVID-19. The Lancet Infectious diseases. 2020;20(6):631. doi: 10.1016/S1473-3099(20)30190-0 32213329 PMC7118670

[85] Xafis V. ‘What is Inconvenient for You is Life-saving for Me’: How Health Inequities are playing out during the COVID-19 Pandemic. Asian Bioethics Review. 2020:1. 10.1007/s41649-020-00119-1 PMC7229879 32427219

[86] Mills AC, Wong-Anuchit C, Poogpan J. A concept analysis of Thum-jai: A Thai coping strategy. Pacific Rim International Journal of Nursing Research. 2017;21(3):234–43.

[87] Mills AC, Poogpan J, Wong‐Anuchit C, Rujkorakarn D. The meaning of acceptance (Thum‐jai) in Thai people: Letting it go… so life goes on. International journal of mental health nursing. 2019;28(4):879–87. doi: 10.1111/inm.12587 30848012

[88] Ross R, Sawatphanit W, Suwansujarid T. Finding Peace (Kwam Sa-ngob Jai) A Buddhist Way to Live With HIV. Journal of Holistic Nursing. 2007;25(4):228–35. doi: 10.1177/0898010106297711 18029962

[89] Dumrongpanapakorn P, Liamputtong P. Social support and coping means: the lived experiences of Northeastern Thai women with breast cancer. Health Promotion International. 2017;32(5):768–77. doi: 10.1093/heapro/dav023 25876908

[90] Liamputtong P, Suwankhong D. Living with breast cancer: the experiences and meaning‐making among women in Southern Thailand. European journal of cancer care. 2016;25(3):371–80. doi: 10.1111/ecc.12321 25899775

[91] Soonthornchaiya R. Resilience for psychological impacts of COVID-19 pandemic on older adults in Thailand. HSOA Journal of gerontology and geriatric medicine. 2020;6:053.

[92] Saud M, Ashfaq A, Abbas A, Ariadi S, Mahmood QK. Social support through religion and psychological well-being: COVID-19 and coping strategies in Indonesia. Journal of religion and health. 2021;60(5):3309–25. doi: 10.1007/s10943-021-01327-1 34245436 PMC8272444

[93] Kowalczyk O, Roszkowski K, Montane X, Pawliszak W, Tylkowski B, Bajek A. Religion and faith perception in a pandemic of COVID-19. Journal of religion and health. 2020;59(6):2671–7. doi: 10.1007/s10943-020-01088-3 33044598 PMC7549332

[94] Sueca IN, Sumertha IW, Winaja IW. A time-lag study on perceived threat of COVID-19 in Hindu religious community: Moderating role of Hindu religious coping. Journal of Ethnic and Cultural Studies. 2021;8(3):217–43.

[95] Ting RS-K, Yong Y-YA, Tan M-M, Yap C-K. Cultural responses to COVID-19 pandemic: Religions, illness perception, and perceived stress. Frontiers in psychology. 2021;12. 10.3389/fpsyg.2021.634863 PMC8375556 34421700

[96] Ironson G, Lucette A, Hylton E, Pargament KI, Krause N. The relationship between religious and psychospiritual measures and an inflammation marker (CRP) in older adults experiencing life event stress. Journal of religion and health. 2018;57(4):1554–66. doi: 10.1007/s10943-018-0600-8 29594652

[97] Phelps AC, Maciejewski PK, Nilsson M, Balboni TA, Wright AA, Paulk ME, et al. Religious coping and use of intensive life-prolonging care near death in patients with advanced cancer. JAMA. 2009;301(11):1140–7. doi: 10.1001/jama.2009.341 19293414 PMC2869298

[98] Baniamin HM, Rahman M, Hasan MT. The COVID-19 pandemic: why are some countries coping more successfully than others? Asia Pacific Journal of Public Administration. 2020;42(3):153–69.

[99] Badshah SL, Ullah A. Spread of coronavirus disease-19 among devotees during religious congregations. Annals of Thoracic Medicine. 2020;15(3):105. doi: 10.4103/atm.ATM_162_20 32831930 PMC7423201

[100] Pfeiffer J, Li H, Martez M, Gillespie T. The role of religious behavior in health self-management: A community-based participatory research study. Religions. 2018;9(11):357.

[101] Phillips Ii G, Felt D, Ruprecht MM, Wang X, Xu J, Perez-Bill E, et al. Addressing the Disproportionate Impacts of the COVID-19 Pandemic on Sexual and Gender Minority Populations in the United States: Actions Toward Equity. LGBT Health. 2020;7(6):279–82. Epub 2020/08/14. doi: 10.1089/lgbt.2020.0187 . 32790495 PMC8106250

[102] Shadmi E, Chen Y, Dourado I, Faran-Perach I, Furler J, Hangoma P, et al. Health equity and COVID-19: global perspectives. Int J Equity Health. 2020;19(1):104. Epub 2020/06/27. doi: 10.1186/s12939-020-01218-z ; PubMed Central PMCID: PMC7316580. 32586388 PMC7316580

[103] The World Bank. Educational attainment, at least completed short-cycle tertiary, population 25+, total (%) (cumulative)—Thailand 2019 [26.04.2022]. Available from: https://data.worldbank.org/indicator/SE.TER.CUAT.ST.ZS?locations=TH.

[104] Jarrett BA, Peitzmeier SM, Restar A, Adamson T, Howell S, Baral S, et al. Gender-affirming care, mental health, and economic stability in the time of COVID-19: a global cross-sectional study of transgender and non-binary people. MedRxiv. 2020. doi: 10.1101/2020.11.02.20224709 34242317 PMC8270151

[105] Gooren LJ, Sungkaew T, Giltay EJ, Guadamuz TE. Cross-sex hormone use, functional health and mental well-being among transgender men (Toms) and Transgender Women (Kathoeys) in Thailand. Culture, health & sexuality. 2015;17(1):92–103. 10.1080/13691058.2014.950982 PMC4227918 25270637

[106] Hensen B, Mackworth-Young C, Simwinga M, Abdelmagid N, Banda J, Mavodza C, et al. Remote data collection for public health research in a COVID-19 era: ethical implications, challenges and opportunities. Health policy and planning. 2021;36(3):360–8. doi: 10.1093/heapol/czaa158 33881138 PMC7928874

[107] Economist Intelligence Unit. EIU Democracy Index 2019—World Democracy Report.

[108] International Labour Organization. International Standard Classification of Occupations 08 (ISCO-08) 2010 [cited 2021 15 January 2021]. Available from: http://www.ilo.org/public/english/bureau/stat/isco/index.htm.

